# *Tetracarpidium conophorum* nuts (African walnuts) up‐regulated adiponectin and PPAR‐γ expressions with reciprocal suppression of TNF‐α gene in obesity

**DOI:** 10.1111/jcmm.70086

**Published:** 2024-12-19

**Authors:** Grace Ufedo Umoru, Item Justin Atangwho, Esien David‐Oku, Daniel Ejim Uti, Eyuwa Ignatius Agwupuye, Uket Nta Obeten, Swastika Maitra, Vetriselvan Subramaniyan, Ling Shing Wong, Nada H. Aljarba, Vinoth Kumarasamy

**Affiliations:** ^1^ Department of Biochemistry, College of Sciences Evangel University Akaeze Okpoto Ebonyi State Nigeria; ^2^ Department of Biochemistry, Faculty of Basic Medical Sciences University of Calabar Calabar Nigeria; ^3^ Department of Biochemistry, Faculty of Basic Medical Sciences, College of Medicine Federal University of Health Sciences Otukpo Benue State Nigeria; ^4^ Department of Research and Publications Kampala International University Kampala Uganda; ^5^ Department of Chemistry/Biochemistry and Molecular Biology Alex Ekwueme Federal University Abakaliki Ebonyi State Nigeria; ^6^ Center for Global Health Research, Saveetha Medical College and Hospital Saveetha Institute of Medical and Technical Sciences Chennai India; ^7^ Pharmacology Unit, Jeffrey Cheah School of Medicine and Health Sciences Monash University Bandar Sunway Malaysia; ^8^ Department of Medical Sciences Sunway University Bandar Sunway Malaysia; ^9^ Faculty of Health and Life Sciences INTI International University Nilai Malaysia; ^10^ Department of Biology, College of Science Princess Nourah bint Abdulrahman University Riyadh Saudi Arabia; ^11^ Department of Parasitology and Medical Entomology, Faculty of Medicine Universiti Kebangsaan Malaysia Kuala Lumpur Malaysia

**Keywords:** adiponectin, obesity, PPARγ, *Tetracarpidium conophorum* nuts, TNF‐α

## Abstract

*Tetracarpidium conophorum* nuts are nutrient‐dense Nigerian snacks associated with weight regulation. This study explores the nuts' impact on adipose tissue gene expression associated with low‐grade inflammation. Ethanol whole extract (EWE), ethyl‐acetate fraction (EAF) and the resulting residue (RES) were orally administered once daily to MSG‐induced obese rats for 6 weeks (*n* = 6). Afterward, the RNA synthesis of inflammation‐associated genes was measured, and GC–MS ligands in the extract and fractions were docked against their protein products in silico. The study found that in obese animals, PPAR‐γ and Adiponectin expressions were down‐regulated, while TNF‐α was up‐regulated, indicating an increased low‐grade inflammatory process in adipose tissue. After 6‐week oral treatments with EWE, EAF and RES, PPAR‐γ and Adiponectin expressions increased significantly, while TNF‐α expression decreased, suggesting the modulation of obesity‐induced inflammation in adipose tissue. The in silico molecular docking analysis identified four lead compounds likely responsible for the observed effect, namely 6‐Isopropenyl‐4,8a‐dimethyl‐4a,5,67,8,8a‐hexahydro‐1H‐naphthalen‐2‐one, 9,12,15‐Octadecatrienoic methyl ester (Z,Z,Z), 9,12,15‐Octadecatrienoic acid and Hexanedioic acid, bis(2‐ethylhexyl). Of these compounds, 6‐Isopropenyl‐4,8a‐dimethyl‐4a,5,67,8,8a‐hexahydro‐1H‐naphthalen‐2‐one demonstrated the strongest affinity to the binding cavities of PPARγ (−7.3 kcal/mol), Leptin (−5.2 kcal/mol), Adiponectin (−7.1 kcal/mol) and TNF‐α (−6.3 kcal/mol) and was better than the standard drug, Orlistat (−6.7, −4.4, −6.8 and − 4.5 kcal/mol, respectively). The study reveals that *T. conophorum* nuts possess bioactive compounds/drug candidates that can exert positive modulation, at the molecular level, the low‐grade inflammatory process associated with obesity, which normally facilitates the outset of complications.

## 1 INTRODUCTION

*Tetracarpidium conophorum*is a 10–20 ft. climbing shrub cultivated for its nuts which are consumed as snacks, when in season, in Nigeria and tropical Africa who it is dominant, and hence the common name, African black Walnut. The nuts are known to be endowed with phytonutrients and bioactive compounds such as antioxidants, phenols (elligatannis, antitellimagrandins) and flavonoids.^1^, ^2^ Studies of chemical evaluation of nuts have shown the presence of oxalates, tannins, phytates, alkaloids and terpenoids.^1^, ^2^, ^3^ The nuts are also reported to possess anti‐inflammatory and antioxidant properties relevant in lowering the risk of chronic inflammation and chronic oxidative stress.^1^, ^4^ In an earlier study, on the impact of the African walnuts on oxidative stress markers, we showed that the nuts assuaged peroxidation and oxidative stress in obese rat model^5^ hence a potential natural product for sourcing anti‐obesity therapeutics.

Obesity as defined, is associated with chronic low‐grade inflammation, which is one of the critical processes required for the development of insulin resistance, diabetes and related diseases.^6^ Overweight and obesity is caused by disproportionate accumulation of white adipose tissue which is usually accompanied by a generalized change in the circulating levels of several adipokines. For instance, studies have shown that increased levels of pro‐inflammatory adipokines and decreased levels of anti‐inflammatory adipokines in obesity account for the chronic state of low‐grade inflammation.^7^, ^8^ Against this background, the expression of key pro‐inflammatory factors, Leptin and TNF‐α and anti‐inflammatory proteins namely PPARγ and adiponectin in the adipose can be manipulated in favour of obesity reduction. Moreover, PPARγ transcription factor which regulates key genes of inflammation namely; adiponectin^9^ and TNF‐α^10^ is germane in the control of inflammation in obesity.

The foregoing indicates a possibility of suppressing the complications of obesity by modulation of its associated inflammation. It is in this wise, that the current study was undertaken. The expressions of inflammation associated genes were evaluated after a 6‐week exposure of obese rats to extracts and fractions of the African walnuts and correlated with in silico interaction of their protein products with GC–MS ligands in the African walnut extracts. Previously, the anti‐obesity activity of the nuts was reported using anthropometric and some biochemical data.^5^, ^11^ However, the molecular mechanism of the anti‐obesity properties of the nuts is yet unknown, hence the need for the current study. It sought to understand at least in part, the molecular mechanism of the nuts anti‐obesity activity using the anti‐inflammatory pathway. In so doing, we studied the interaction of the extracts and fractions from the nut with key molecular targets of the inflammatory pathway in vivo and in silico. As a conventional nomenclature, the genes encoding the transcription factors shall be referred to as upstream genes and those whose expression they regulate, as the downstream genes.

In order to better understand the impact of bioactives in African walnuts on markers of low‐grade inflammation associated with obesity, that usually elicit the outset of its comorbidities such as cancers, the study was undertaken. A study such as this, is relevance is sourcing drug compounds from natural products, with comparative advantage over the existing medication or that would serve as adjuncts to the existing medications, contributing to the needed mitigation of the looming global obesity epidemic.

## 2 MATERIALS AND METHODS

### 2.1 Chemicals and reagents

The chemicals and reagents used in this study included Easy Script One‐Step RT‐PCR SuperMix (Beijing TransGen Biotech Co., Ltd., Beijing, China),Primers (Integrated Data Technologies, 802 Old Dixie Hwy #2, North Palm Beach, FL 33408, United States), TRIquick reagent (solarbio life sciences), RNase free water (Transgenbiotec). All other reagents and chemicals used but not listed here were of analytical grade and were purchased from Sigma‐Aldrich, USA, unless otherwise stated.

#### 2.1.1 Plant material

The African walnuts were purchased from Sagamu, Ogun State, brought to the University. The nuts were authenticated by Dr. Bruno Aduo, of the Biological Sciences Department of the same school. The shells were removed and chopped into small bitsand dried at room temperature (25°–29°C). Upon drying they were ground into fine powder and thereafter soaked in ethanol: 2 parts of solvent to 1 part of sample. The suspension was shaken vigorously and left to settle in a jar for 48 h at room temperature after which it went through filtration twice, first, with a cheese material and afterwards with Whatman No. 2 filter paper. The filtrate was placed in a rotary evaporator (45°–50°C) where it was concentrated to about 1/10th of its initial volume and placed in a water bath (45–50°C) for solvent evaporation and drying. Once dry, the weight of extract was taken and it is percent yield calculated as follows:

$$\%\text{Yield}=\frac{\text{Weight of extract}}{\text{Weight of starting material}}\times 100$$

#### 2.1.2 Liquid–liquid fractionation of the crude extract

The ethanol whole extract was subjected to further fractionation using the method described in an earlier study by Utiet al.^5^ A known quantity (10 g) of the crude extract was solubilized in 10 mL and 90 mL of solvents ethanol and ethyl acetate respectively in a separating funnel. The mixture agitated and left to settle for 5–10 min. The result was a separation into two visible layers. The less dense upper layer was carefully decanted into an appropriately labelled beaker hence separating it from the denser layer. This fractionation process was repeated until all the components of the whole extract soluble in the fractionation solvent were presumably collected. The residue was then subjected to another cycle of fractionation with a solvent next in polarity grading. In the present study, ethyl acetate was the only solvent used in fractionation to produce the following fractions: (I) the ethyl acetate fraction and the Residue fraction. Each fraction was concentrated until dry and their respective percentage yields were calculated as follows: 2 kg of the pulverized sample was used as starting material to give 378.56 g of whole ethanol extract representing 18.93% yield. A portion of this extract fractionated with ethyl acetate produced 251.71 g, of ethyl acetate fraction, representing 66.49% yield. The fractions were stored in a freezer at −4°C to be used for animal and phyto‐chemical analysis.

### 2.2 Animal studies

#### 2.2.1 Induction of experimental obesity

Adult Wistar rats (male and female) from the Animal House, College of Medical Sciences University of Calabar, were used. They were placed together three females to one male to breed. Their offspring served as obese animal models according to methods described in our earlier study, Uti et al.^11^ The litters were categorized into two groups on the day of delivery; the first group (group 1) was administered a single dose of 4 mg/g b.w. of MSG dissolved in normal saline via intra‐peritoneal route, on postnatal days 2, 4, 6, 8 and 10, while the second group (group 2) were treated similarly with only normal saline. On the 21st day, the rats were weaned after which they were reared and studied till they were 12 weeks old. Lee's Index and Body Mass Index were used as obesity indicators, and these were calculated as follows:

$${\mathrm{Lee}}^{’}s\;\text{Index}=\frac{\text{Body weights}\;{\left(g\right)}^{1/3}}{\text{Nasal}‐\text{anal length}\;\left(\mathrm{cm}\right)}$$

$$\mathrm{BMI}=\frac{\text{Body weights}\;\left(g\right)}{\text{Nasal}‐\text{anal}\;{\text{length}}^{2}\;\left({\mathrm{cm}}^{2}\right)}$$

The rats with Lee's index ≥0.3 and above were considered obese and/or with BMI of 20% greater than controls were considered obese.^12^

#### 2.2.2 Experimental design and procedures

The experimentally obese animals were grouped into five sections of six (6) rats plus a normal control comprise of six rats and treated as shown in the schedule on Table 1. Rat pellets and tap water were given to the animals ad libitum. They were also maintained in a facility of normal room temperature of 25°C ± 3°C. Following a 6‐week treatment period, the animals were humanely euthanized using a widely accepted pharmacological method. This involved administering an injectable overdose of anaesthesia, specifically ketamine (2 μL/kg b.w.), via intraperitoneal injection. As a result of this anaesthesia, the rats quickly lost consciousness. Subsequently, the rats were fully exsanguinated by performing a heart puncture using hypodermic needles and syringes. Following this procedure, the animals underwent sterile dissection, during which their livers and epididymal adipose tissues were carefully excised and placed into sample bottles containing RNA later. These samples were then frozen at −80°C until they were ready for analysis. It is important to note that all procedures involving the animals were conducted in accordance with the guidelines outlined in the National Institute of Health (NIH) publication from 1985, which pertains to the care and use of laboratory animals. Furthermore, ethical clearance for the use of animals in this study was obtained from the Faculty of Basic Medical Sciences Animal Research Ethics Committee (FAREC‐FBMS) at the University of Calabar, Nigeria, with the approval number 032BCH3319.

**TABLE 1:** Experimental groups of obese rats treated with *T. conophorum* nut extracts and fractions.

S/N	Group	Treatment
1	Normal control (NC)	Normal saline
2	Obese control (OC)	Normal saline
3	Standard control (SC)	5.14 mg/kg b.wt Orlistart
4	Ethanol whole extract (EWE)	2 g/kg b.wt of EWE
5	Ethyl acetate fraction (EAF)	2 g/kg b.wt of EAF
6	Residue fraction (RES)	2 g/kg b.wt of RES

#### 2.2.3 Homogenization and RNA extraction

Tissue homogenization and RNA extraction was carried out according to the earlier procedures of Atangwho, Yin.^13^ Briefly, 500 mL of Trizol reagent was added to the samples and homogenized. In the Trizol solution containing the disrupted tissue, 100 mL of chloroform was added and shaken vigorously for 30 s, then allowed to stand for 10 min. The mixture was centrifuged at 11,300 rpm for 15 min to separate into three layers, and the clear liquid layer containing the RNA carefully aspirated into new tubes, as the RNA extract. The RNA in the extract was precipitated by addition of cold isopropyl alcohol (0.25 mL), vortexed and left to settlefor 10 min at room temperature. Thereafter, the suspension was centrifuged at 12,000 rpm for 10 min and the top layer was decanted leaving the residue, the recovered RNA. The RNA pellet was re‐washed with 500 μL of cold 75% ethanol (Sigma‐Aldrich) and the tubes were shaken by inversion to softly wash the RNA. The tubes were again centrifuged at 9100 rpm for 5 min. The supernatant was decanted carefully with a pipette, and the pellet recovered and dried in a Centrivap for about 10–15 min. Thereafter, the pellets were re‐solubilized in 50 μL of RNase free water and stored at −20^ᴼ^C.

#### 2.2.4 Determination of purity, concentration and integrity of RNA

The purity of the extracted RNA was determined using a Nano‐spectrophotometer (Nano drop 2000, Thermo Fisher Scientific Nano Drop 2000 spectrophotometer 3411 Silverside Road, Bancroft Building Wilmington, DE 19810 USA) at 260/280 nm. The RNA extracts were considered pure at 260/280 nm ratio between 1.8 and 2.2.^14^ The concentration of the extract was determined directly from the nano‐spectrophotometer.

#### 2.2.5 Complementary DNA (cDNA) synthesis and amplification by conventional polymerase chain reaction (PCR)

Reverse transcription of total RNA was carried out using the TransgenEasyScript® one‐step RT‐PCR supermix (Beijing Trans Gen Biotech Co., Ltd., Beijing, China) according to the manufacturer's instructions. In total, the volume (20 μL) for one conventional PCR reaction contains: 0.4 μL Enzyme Mix, 0.4 μL each of the forward and reverse primers (Table 2), 10 μL reaction mix, 5 μL RNA sample and 3.8 μL of nuclease free water. The PCR reaction was performed in triplicate using Bio‐rad c1000 touch thermo‐cycler with the cycle process shown in Table 3. The results were compared with reference/house‐keeping gene glyceraldehyde‐3‐phosphate dehydrogenase for adipose tissue.

**TABLE 2:** Sequence of gene specific primers.

Gene	Primer	Sequence (5′–3′)	Length
PPAR‐γ	Primer F Primer R	GGG TGC AGC GCT AAA TTC AT CCA AAC CTG ATG GCA TTG TCA	20 22
LEPTIN	Primer F Primer R	GCC AAC GCA AAC CCA TTC TG GAT ACC GAC TGC GTG TGT GA	20 20
ADIPONECTIN	Primer F Primer R	CTC AGG AGA CCT GGC GAT TT TTA GGA CCA AGA ACA CCT GCG	20 21
TNF‐α	Primer F Primer R	GAT CGG TCC CAA CAA GGA GG CAA AGG CGG AGA TGA GAC CC	20 20
GAPDH	Primer F Primer R	GAT GGC CTT CTA CCC GAA GAC GCG GAT GTC ATC CAC TGT TGA	21 21

**TABLE 3:** Cycle parameters.

Temperature	Time	Function
45^o^c	30 min	cDNA synthesis
93^o^c	1 min	Reverse transcriptase inactivation
94^o^c	30 s	Initial denaturation
50^o^c	30 s	Annealing temperature
72^o^c	1 min	Extension
72^o^c	10 min	Final extension
4^o^c	Infinite	Infinite hold

#### 2.2.6 Gel electrophoresis

The amplicons gotten from the PCR process were detected and quantified using agarose gel electrophoresis. In this study, 1.5 g of agarose was solubilized in 100 mL Tris‐borate‐EDTA (TBE) buffer,^15^ stirred, heated and allowed to cool for 5 min. Thereafter, 4 μL of Ethidium bromide was prepared and gently poured into the molten gel, mixed and poured on a gel cast with a comb and allowed to set. The comb when removed created wells into which the samples were applied and allowed to solidify for 30 min at room temperature.^16^ With the solid gel placed in the electrophoresis tank and submerged in 1.0% TBE buffer, the RT‐PCR amplicons were gently pipetted into the wells as well as the blank, and the electrophoresis ranfor 30 min at 95 V and 400 mA. At the end of electrophoresis, gels were placed under a UV trans‐illuminator for detection and examination. The concentrations of the bands were determined using the Image J software (NCBI).

#### 2.2.7 Amplicon interpretation

The cDNA gels were interpreted using ImageJ software^17^ and the data presented as the gene expression relative to the reference genes (housekeeping genes), GAPDH (for adipose tissue extract).

### 2.3 In silico docking analysis

Using PyRx 0.8 software all the ligands from Gas chromatography–mass spectroscopy of the *T. conophorum* were docked with PPARγ, adiponectin and TNF‐α in other to determine their binding energies (binding affinities) towards these proteins.

#### 2.3.1 Preparation of target protein

The crystal structures of PPARγ (PDB ID: 1ZGY), TNF‐α (5WUX), leptin (1AX8) and adiponectin (4DOU) proteins were retrieved from the Protein Data Bank's structural base (https://www.rcb.org/).^18^ First, the active sites of the proteins were determined using Computed Atlas of Surface Topography of Proteins (CASTP)^19^ and proteins separated from their ligands, all hetero atoms and crystal water molecules were removed while hydrogen atoms and gasteiger charges were added to the protein. Finally, the structure was minimized using the software UCSF chimera 1.4 and saved in PDBQT format for further analysis.

#### 2.3.2 Ligand preparation

The crystal structures of chemical profile of *T. conophorum* nut (Table 4) as earlier detected by^11^ were retrieved from the PubChem database (pubchem.ncbi.nlm.nih.gov/compound) in structure data file (sdf) format, imported into the PyRx 0.8 software and subjected to minimization. The energy minimization was performed with the universal force field (UFF) using conjugate gradient algorithm^20^, the minimized compounds were converted to PDBQT format for further analysis.

**TABLE 4:** *T. conophorum* nut derived compounds (ligands) and their PUBCHEM ID.

S/N	COMPOUND	PubChem ID	EWE	EAF	RES
1	Decanoic acid, ethyl ester	8048	Yes	Yes	‐
2	Dodecanoic acid, ethyl ester	7800	Yes	Yes	‐
3	Tetradecanoic acid, ethyl ester	31283	Yes	Yes	‐
4	Hexadecanoic acid methyl ester	8181	‐	Yes	‐
5	Hexadecanoic acid, ethyl ester	12366	Yes	Yes	Yes
6	n‐Hexadecanoic acid	985	‐	Yes	Yes
7	9,12‐Octadecadienoic acid (Z,Z)‐methyl ester	5284421	Yes	‐	Yes
8	9,12‐Octadecadienoic acid, methyl ester	5284421	Yes	Yes	‐
9	Methyl stearate	8201	Yes	Yes	‐
10	9,12,15‐Octadecatrienoic methyl ester (Z,Z,Z)	5364049	Yes	Yes	Yes
11	9,12‐Octadecadienoic acid, ethyl ester	5365672	‐	‐	Yes
12	Ethyl 9,12,15‐octadecatrienoate	5367460	‐	Yes	‐
13	Ethyl 9,12,15‐octadecatrienoate 9,12,15‐Octadecatrienoic acid ethyl ester	5367460	‐	‐	Yes
14	9,12,15‐Octadecatrienoic acid ethyl ester	6371716			
15	9,12,15‐Octadecatrienoic acid (Z,Z,Z)	5282822	Yes	Yes	Yes
16	9,12‐Octadecadienoic acid, ethyl ester	5365672	‐	‐	Yes
17	Linoleic acid ethyl ester	5282184	‐	‐	Yes
18	Octadecanoic acid, ethyl ester	8122	Yes	Yes	Yes
19	2,3‐Dihydroxypropyl elaidate	5364833	‐	‐	Yes
20	cis‐11‐Eicosenoic acid, methylester	5463047	Yes	Yes	‐
21	Ethyl 9‐hexadecenoate	5364759	‐	Yes	‐
22	Methyl 19‐methyl‐eicosanoate	4580601	‐	Yes	‐
23	Hexanedioic acid, bis(2‐ethylhexyl)	7641	Yes	Yes	‐
24	9,12,15‐Octadecatrienoic acid (Z,Z,Z)	5282822	‐	Yes	‐
25	9‐Octadecenoic acid, €‐ Heptasiloxane, 1,1,3,3,5,5,7,7,9911,11,13,13‐tetradecamethyl‐	25202007	Yes	‐	‐
26	9‐Octadecenoic acid (Z)‐, 2,3‐dihy droxypropyl ester	965	‐	‐	Yes
27	8‐Methyl‐6‐nonenamide 13‐Docosenamide, (Z)‐	5365367	Yes	‐	‐
28	6‐Isopropenyl‐4,8a‐dimethyl‐4a,5,6 7,8,8a‐hexahydro‐1H‐naphthalen‐2‐one	565648	Yes	‐	‐
29	Orlistat	3034010	‐	‐	‐

*Source*: Uti et al., 2020.

#### 2.3.3 Virtual screening

The protein crystal structures were used as receptors and the crystal structures of chemical profile of *T. conophorum* nuts as ligands. The binding energies were calculated using PyRx0.8, a gridbox was set to span the active site of the receptor with the dimensions shown in Table 5. An exhaustiveness of eight was used and the top three hit compounds were selected for screening. The top three hit compounds were passed through a second screening using AutoDock 4.2^20^ and analysis of the findings was performed using Discovery studio and PyMOL programs.^21^

**TABLE 5:** Gridbox dimension.

S/N	PROTEIN	PDB ID	GRID BOX LENGTH (Angstrom)		
			*x*‐axis	*y*‐axis	*z*‐axis
1	Peroxisome Proliferated Activator Gamma (PPAR‐γ)	1ZGY	35.23	37.05	41.04
2	Leptin	1AX8	43.80	50.56	58.10
3	Adiponectin	4DOU	29.78	24.79	36.00
4	Tumour Necrosis Factor Alpha (TNF‐α)	2AZ5	15.09	16.06	12.39

#### 2.3.4 Absorption distribution metabolism excretion and toxicity (ADMET)/pharmacokinetics predictions analysis

Absorption Distribution Metabolism Excretion and Toxicity (ADMET) analysis makes up the pharmacokinetics of a drug molecule. Here, the molecular structure of top three HIT ligands were submitted to Swissadme (http://www.swissadme.ch) and admetSAR server (http://lmmd. ecust.edu.cn:8000) to examine their drug likeliness using the Lipinski rule of five which states that a molecule or an inhibitor can be orally absorbed/active if two (2) or more of these thresholds; molecular weight (Mw) of molecule <500, octanol/water partition coefficient (iLOGP) ≤ 5, number of hydrogen bond acceptors (nHBA) ≤ 10, number of hydrogen bond donors (nHBD) ≤ 5, and topological polar surface area (TPSA) <40 Å2 are not violated. Different pharmacokinetic and pharmacodynamic parameters (mutagenicity, blood brain barrier penetration, human intestinal absorption, cytochrome P450 solubility, cytochrome P (CYP) inhibitory promiscuity and Ames toxicity) were also analysed.

#### 2.3.5 Statistical analysis

The results were expressed as the Mean ± Standard Deviation (SD), and the differences between treated and control groups were statistically analyzed using one‐way ANOVA and paired with one sample *t*‐test. All statistical calculations were performed using GRAPHPAD prism7.0 (GraphPad Software, CA, USA) followed by post hoc *Turkey multiple range test and LSD*. The results were considered significant at *p* < 0.05.

## 3 RESULTS

### 3.1 Effect of the extract and fractions on the expression of the regulatory target (upstream) gene, PPARγ in the adipose tissue

**FIGURE 1:**
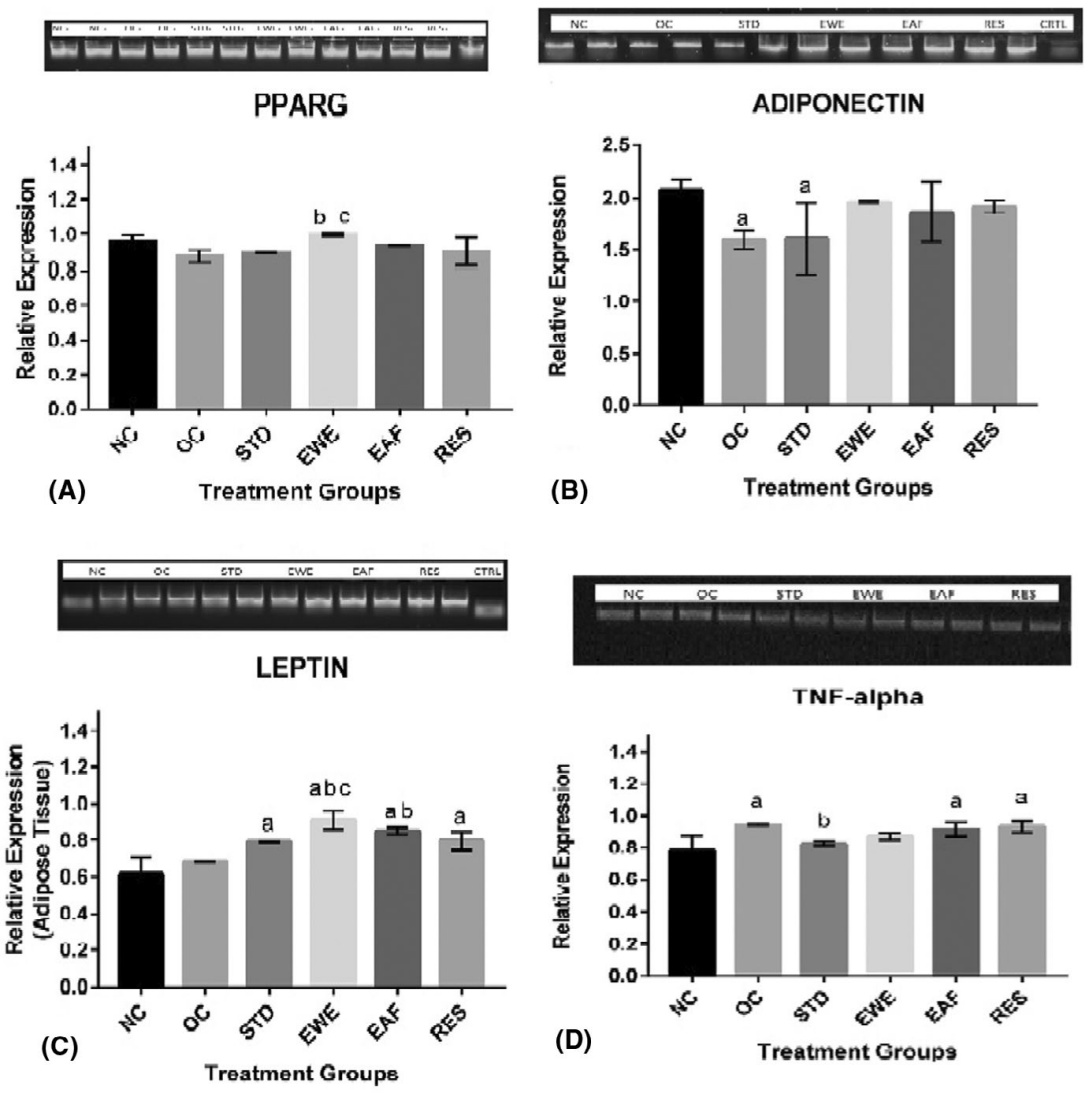
(A) Relative expression of PPARγ (B) Relative expression of adiponectin, (C) Relative expression of Leptin and (D) Relative expression of TNF‐alphain the adipose tissue; of monosodium glutamate‐induced obese rats administered *Tetracarpidium conophorum* nut extracts and fractions. NC, Normal control; OC, Obese control; STD, Standard control; WE, Whole extract; ET, Ethyl‐acetate fraction; RES, Residue fraction; b, *p* < 0.05 versus OC; c, *p* < 0.05 versus STD; a, significantly different from NC at *p* < 0.05 *n* = 5.

Figure1A shows result of the relative expressions of PPARγ in the adipose tissue of obese rats treated with the nut extract and fractions. There was observed down‐regulation in the expression of PPARγ by 8.9% in obese rats compared to the normal control (*p* < 0.05). However, 6‐week treatment with EWE, EAF and RES was found to up‐regulate the PPARγ expression by 12.0%, 7.6% and 3.9% relative to the obese control (*p* < 0.05).

### 3.2 Effect of the extract and fractions on the expressions of select inflammation associated genes(leptin, adiponectin and TNF‐α) in the adipose

Figure 1B–D show the respective relative expressions of adiponectin, leptin and TNF‐αgenes in the adipose tissue of the study animals—treated versus controls: Adiponectin expression decreased in the obese control compared to the normal control. This was increased after 6‐week administration of EWE, EAF and the RES by 19.1%, 13.9% and 16.9% respectively, relative to the obese control (*p* < 0.05). Also, TNF‐α gene expression that was up‐regulated in the obese control, was found to decrease at the end of study by the EWE only (8.9%). The EAF and RES failed to exert significant effect, suggesting that fractionation may have rendered ineffective, a possible synergistic effect of the bioactive compounds. The leptin gene expression was only mildly effected by the nut extracts and fractions.

### 3.3 In silico studies of regulatory target (upstream) gene product, PPARγ

**FIGURE 2:**
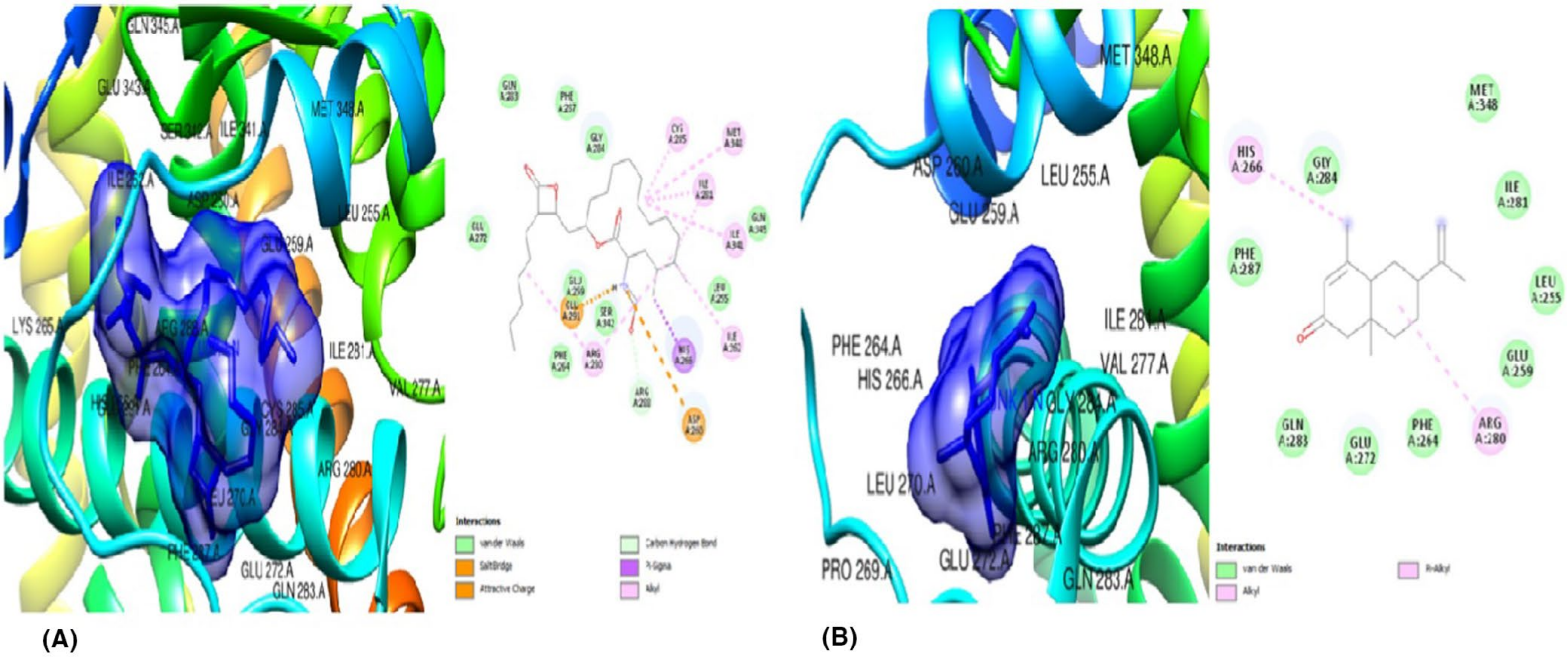
(A) 3D and 2D docked structures of PPARγ (1ZGY) complexed with Orlistat (Binding energy −56.7 kcal/mol). (B) 3D and 2D docked structure of PPARγ (1ZGY) complexed with 6‐Isopropenyl‐4,8a‐dimethyl‐4a,5,6 7,8,8a‐hexahydro‐1H‐naphthalen‐2‐one (Binding affinity −7.3 kcal/mol).

**FIGURE 3:**
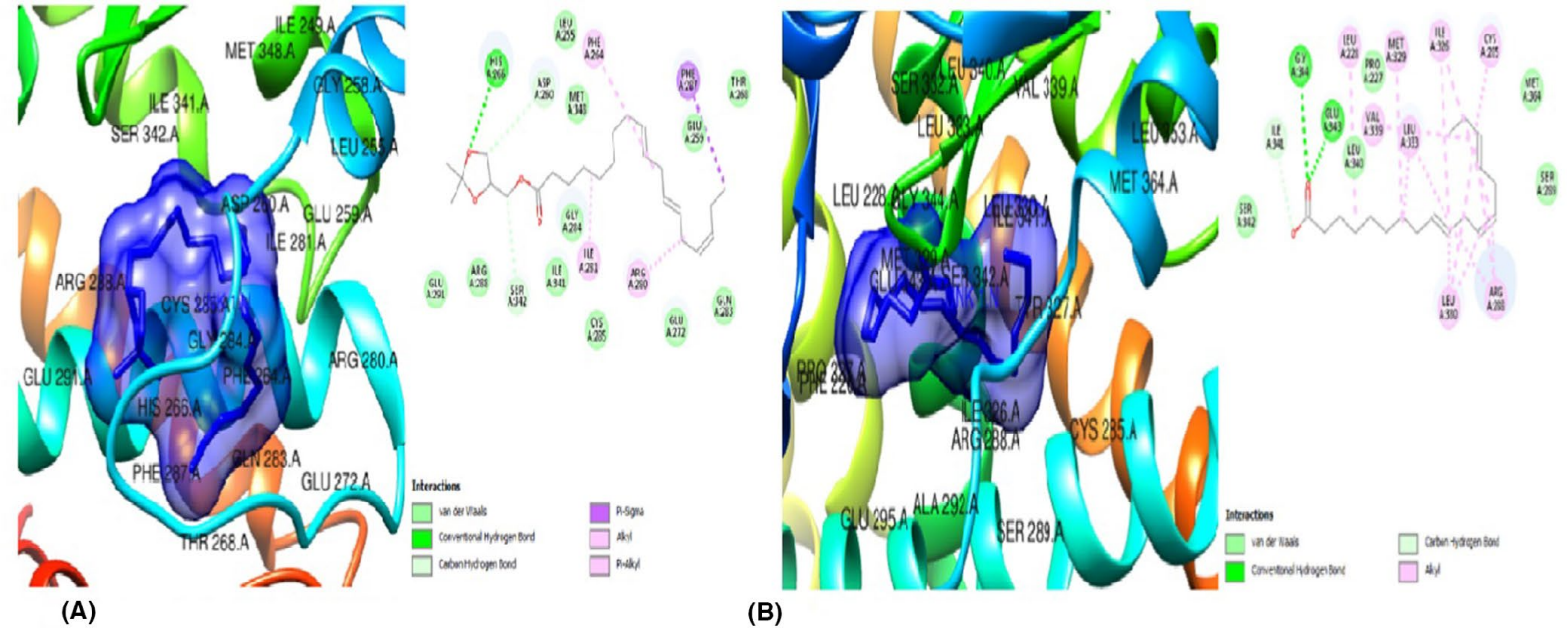
(A) 3D and 2D docked structures of PPARγ (1ZGY) complexed with 9,12,15‐Octadecatrienoic methyl ester (Z,Z,Z) (Binding affinity −6.8 kcal/mol). (B) 3D and 2D docked structures of PPARγ (1ZGY) complexed with 9,12,15‐Octadecatrienoic acid (Z,Z,Z) (Binding energy −6.6 kcal/mol).

The ligands from *T. conophorum* nuts were docked against the target protein, PPARγ (1ZGY) and the resulting binding affinities shown in Figures 2 and 3. The top three hit poses with the lowest binding energies and highest binding affinities compared to the standard drug Orlistat (Figure 2A) were: (i) 6‐Isopropenyl‐4,8a‐dimethyl‐4a,5,6,7,8,8a‐hexahydro‐1H‐naphthalen‐2‐one (Figure 2B) with binding affinity of −7.3Kcal/mol, nine (|9) van der Waal forces, one (1) alkyl and one (1) pi‐alkyl bonds. Interactions of this ligand with the PPARγ protein were via His 266 and Arg 280 residues. (ii) 9,12,15‐Octadecatrienoic methyl ester (Z,Z,Z) (Figure 3A) with −6.8Kcal/mol binding energy/affinity, eleven (11) van der Waal interactions, one (1) conventional hydrogen bond, two (2) carbon hydrogen bonds, one (1) pi‐sigma bond, one (1) alkyl and two |(2) pi‐alkyl bonds. This second ligand interacted with the PPARγ protein via His 266, Asp 260, Ser 343, Phe 264, Ile 281, Arg 280 and Phe 287 residues. (iii) 9,12,15‐Octadecatrienoic acid, (Z,Z,Z) (Figure 3B)with the highest binding energy of −6.7Kcal/mol and docking characteristics of five (5) van der waal forces, two (2) conventional hydrogen bonds, one (1) carbon hydrogen bond and eight (8) alkyl bonds. The residues Ile 341, Gly 344, Glu 343, Leu 228, Met 329, Val, 339, Leu 333, Ile 326, Leu 330, Cys 285 and Arg 288 were found to be involved its interaction with the PPARγprotein.

#### 3.3.1 In silico studies of the products of selected inflammatory genes of the adipose (Leptin, Adiponectin and TNF‐alpha)

**FIGURE 4:**
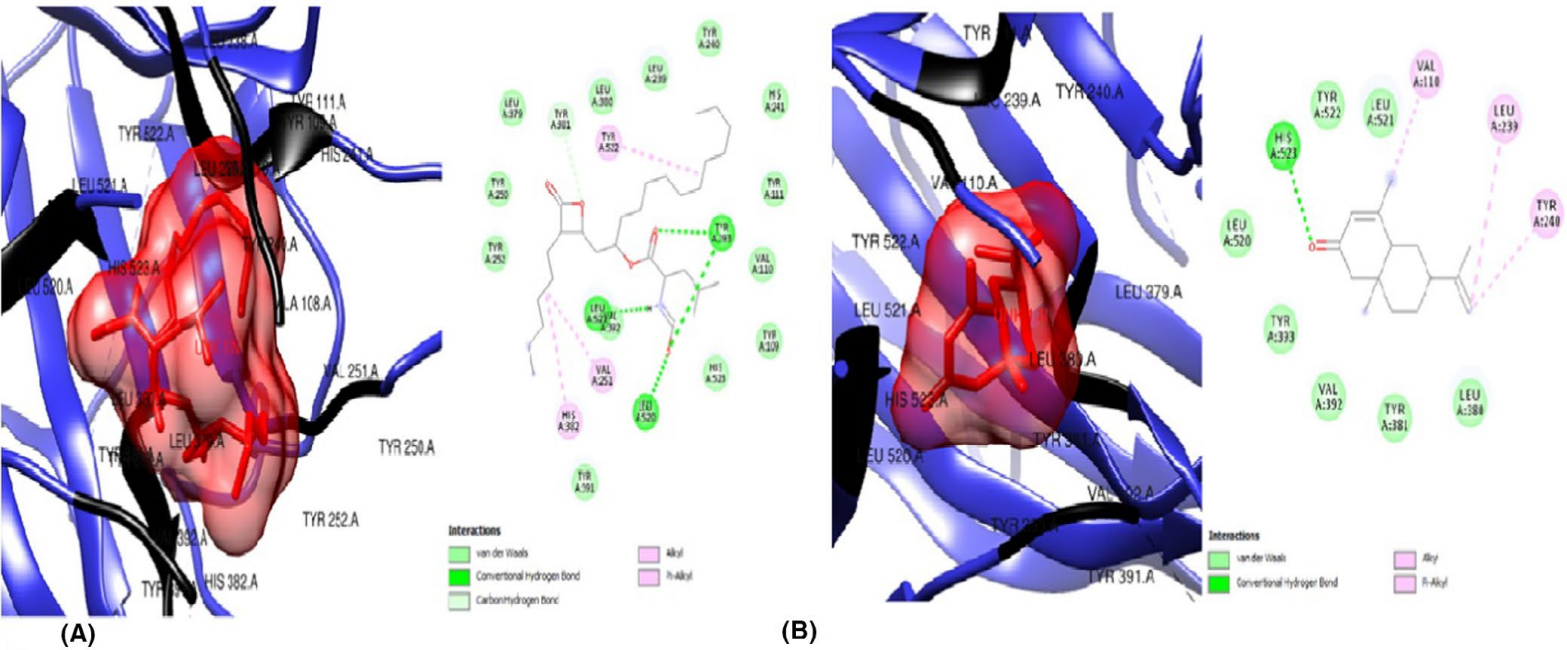
(A) 3D and 2D docked structures of ADIPONECTIN (4DOU) complexed with Orlistat (Binding affinity −6.8 kcal/mol). (B) 3D and 2D docked structures of ADIPONECTIN (4DOU) complexed with 6‐Isopropenyl‐4,8a‐dimethyl‐4a,5,6 7,8,8a‐hexahydro‐1H‐naphthalen‐2‐one (Binding affinity −7.1 kcal/mol).

**FIGURE 5:**
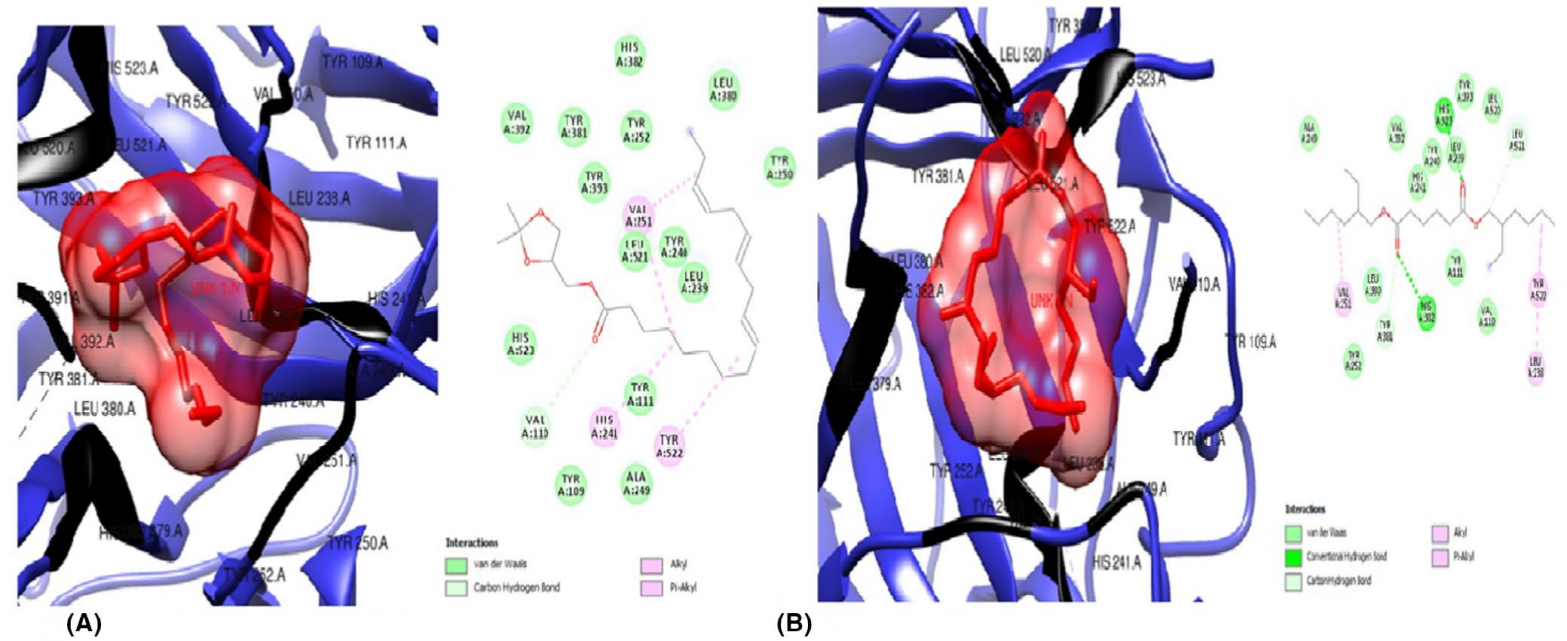
(A) 3D and 2D docked structures of ADIPONECTIN (4DOU) complexed with 9,12,15‐Octadecatrienoic methyl ester (Z,Z,Z) (Binding affinity −6.7 kcal/mol). (B) 3D and 2D docked structures of ADIPONECTIN (4DOU) complexed with Hexanedioic acid (Binding affinity −6.1 kcal/mol).

The GC–MS bio‐actives/ligands of *T. conophorum* nuts were also docked against the anti‐inflammatory protein, adiponectin(4DOU) and results of the top three hit compounds along with the standard drug Orlistat (Figure 4A)are shown on Table 6. Of the three compounds, 6‐Isopropenyl‐4,8a‐dimethyl‐4a,5,6,7,8,8a‐hexahydro‐1H‐naphthalen‐2‐one (Figure 4B)exerted the highest binding affinity (−7.1 kcal/mol) compared to the standard drug, Orlistat (−6.8 kcal/mol). Moreover, it possessed seven (7) van der Waals forces, one (1) conventional hydrogen bond, two (2) alkyl bonds, one (1) pi‐alkyl bond and interacted with the protein target via His 523, Val 110, Leu 239 and Tyr 240 residues. This ligand was followed closely by 9,12,15‐Octadecatrienoic methyl ester (Z,Z,Z) (Figure 5A) with a binding affinity of −6.7 kcal/mol, fourteen (14) van der Waal forces, one (1) carbon hydrogen bond, two (2)alkyl and one (1) pi‐alkyl bond. The residues Val 251, Val 110, His 241 and Tyr 522 were found to be involved in its interaction with the target protein. The ligand with the least binding affinity of the three compounds is Hexanedioic acid (Figure 5B) with‐6.1 kcal/mol, eleven (11)van der Waal linkages, two (2) conventional hydrogen bonds, two(2) carbon hydrogen bonds, two (2) alkyl and one(1) pi‐alkyl bonds. These interactions were via the Val 251, His 523, Leu 521, Tyr 522 and Leu 238 residues.

**TABLE 6:** Showing binding energies and number of hydrogen bonds of hit compounds.

		PPAR‐γ	Leptin	Adiponectin	TNF‐α
		BE (Kcal/mol)	BE (Kcal/mol)	BE (Kcal/mol)	BE (Kcal/mol)
Orlistat	3034010	−6.7	−4.4	−6.8	−4.5
6‐Isopropenyl‐4,8a‐dimethyl‐4a,5,67,8,8a‐hexahydro‐1H‐naphthalen‐2‐one	565648	−7.3	−5.2	−7.1	−6.3
9,12,15‐Octadecatrienoic methyl ester (Z,Z,Z)	5364049	−6.8		−6.7	−5.1
9,12,15‐Octadecatrienoic acid (Z,Z,Z)	5282822	−6.6			−5.0
Hexanedioic acid, bis(2‐ethylhexyl)	7641		−4.6	−6.1	
9‐ Octadecenoic acid			−4.6		

Abbreviation: BE, Binding energy.

**FIGURE 6:**
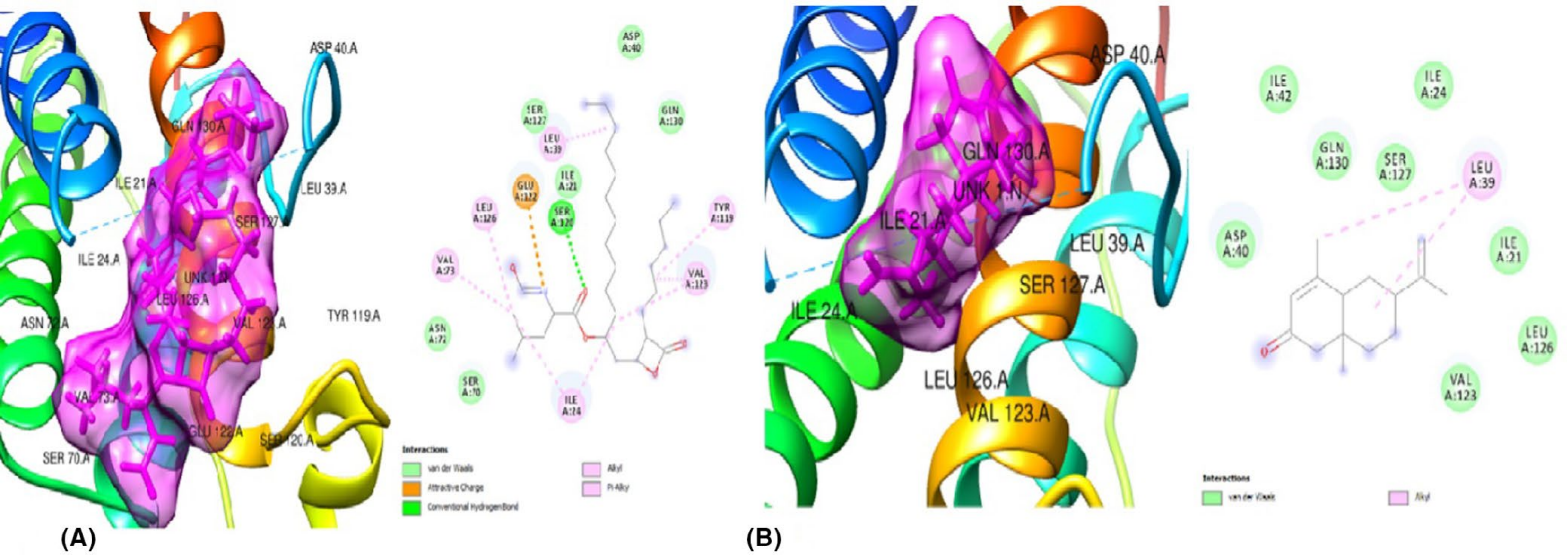
(A) 3D and 2D docked structures of LEPTIN (1AX8) complexed with Orlistat (Binding affinity −4.4 kcal/mol). (B) 3D and 2D docked structures of LEPTIN (1AX8) complexed with 6‐Isopropenyl‐4,8a‐dimethyl‐4a,5,6 7,8,8a‐hexahydro‐1H‐naphthalen‐2‐one (Binding affinity −5.2 kcal/mol).

**FIGURE 7:**
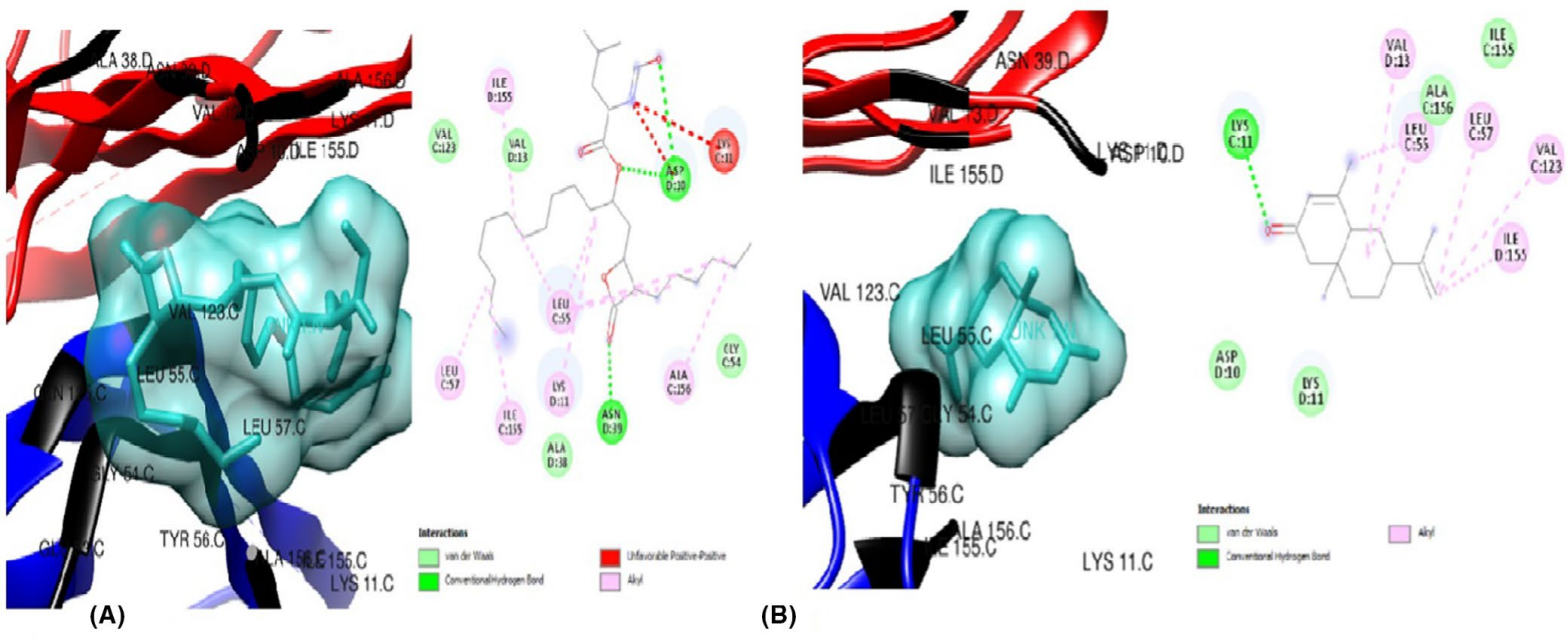
(A) 3D and 2D docked structures of LEPTIN (1AX8) complexed with Hexanedioic acid, bis(2‐ethylhexyl) (Binding affinity −4.6 kcal/mol). (B) 3D and 2D docked structures of LEPTIN (1AX8) complexed with 9‐Octadecenoic acid (Binding affinity −4.6 kcal/mol).

**FIGURE 8:**
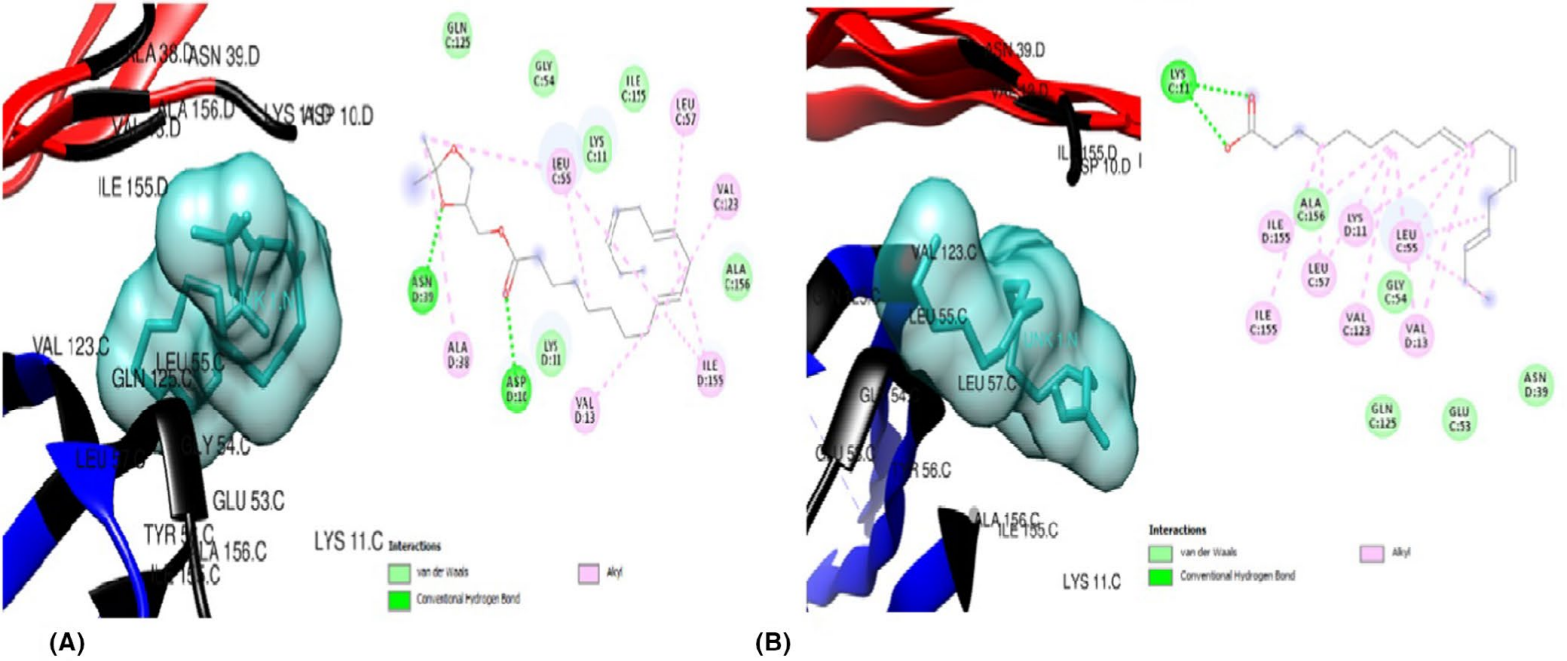
(A) 3D and 2D docked structures of TNF‐alpha (5WUX) complexed with Orlistat (Binding affinity −4.5 kcal/mol). (B) 3D and 2D docked structures of TNF‐alpha (5WUX) complexed with 6‐Isopropenyl 4,8a‐dimethyl‐4a,5,6 7,8,8a‐hexahydro‐1H‐naphthalen‐2‐one (Binding affinity −6.3 kcal/mol).

**FIGURE 9:**
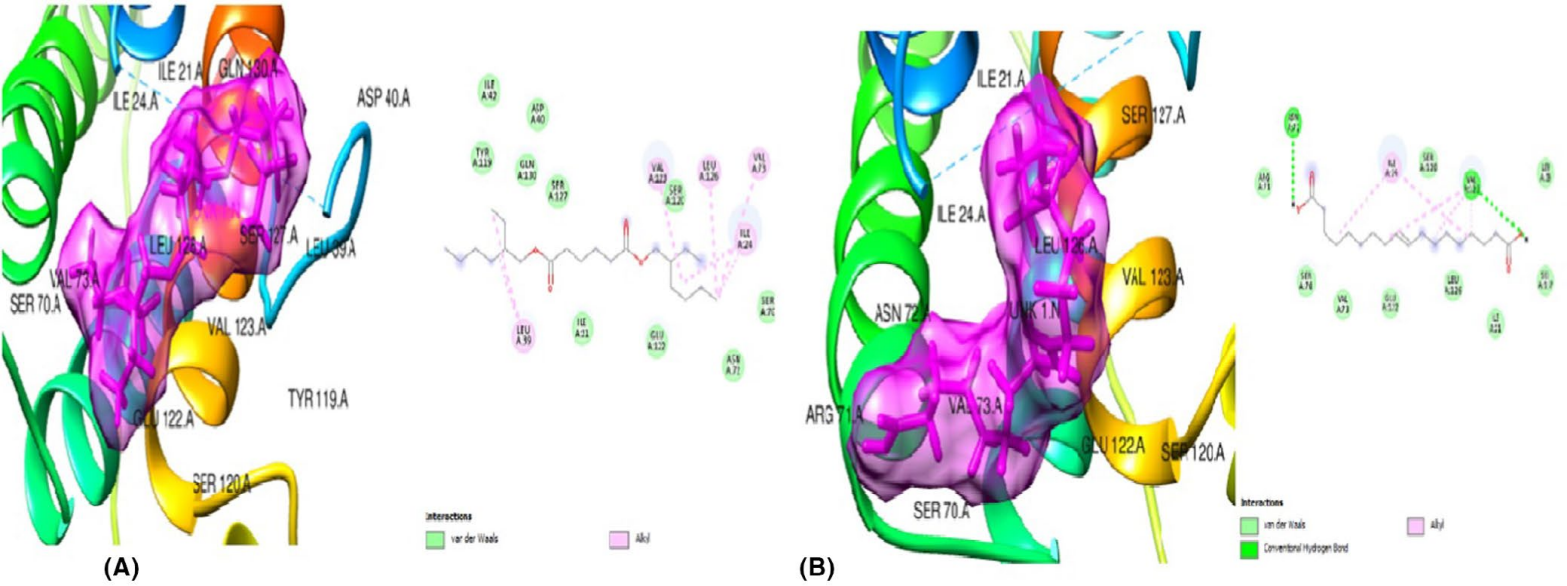
(A) 3D and 2D docked structures of TNF‐alpha (5WUX) complexed with 9,12,15‐Octadecatrienoic methyl ester (Z,Z,Z) (Binding affinity −5.1 kcal/mol). (B) 3D and 2D docked structures of TNF‐alpha (5WUX) complexed with 9,12,15‐Octadecatrienoic acid (Z,Z,Z) (Binding affinity −5.0 kcal/mol).

The ligands were also docked against the pro‐inflammatory target proteins, Leptin (1AX8) and TNF‐α (5WUX). The top poses for the top three compounds with the highest binding affinity against Leptin compared to the standard drug, orlistat were: 6‐Isopropenyl‐4,8a‐dimethyl‐4a,5,6,7,8,8a‐hexahydro‐1H‐naphthalen‐2‐one (Figure 5B) which exerted the lowest binding energy of −5.5 kcal/mol and highest affinity for leptin, eight (8) van der Waal forces, one alkyl bond and interacts with only residue Leu 39. The second most efficacious was hexanedioic acid bis (2‐ethylhexyl) (Figure 6A) with binding energy of −4.6 kcal/mol, ten (10) van der waal forces and five (5) alkyl bonds, interacting with the residues Leu 39, Ser 120, Leu 126, Val 73 and Ile 24. Lastly, 9‐octadecenoicacid (Figure 6B)with binding energy of −4.6 kcal/mol, two (2) conventional hydrogen bonds, nine (9) van der Waal forces and one (1) alkyl bond. The residues Asn 72, Ile 24 AND Val 123 were involved in this interaction. In docking against TNF‐α, the three hit compoundswere6‐Isopropenyl‐4,8a‐dimethyl‐4a,5,6,7,8,8a‐hexahydro‐1H‐naphthalen‐2‐one (Figure 7B), which showed the lowest binding energy of −6.3 Kcal/moland highest affinity for TNF‐α, four (4) van der Waal forces, one (1) conventional hydrogen bond and five (5) alkyl bonds; and interacted with the residues Lys 11, Val 13, Leu 55, Leu 57, Val 123 and Ile 155. The next most striking interacting ligand was 9,12,15‐Octadecatrienoic methyl ester (Z,Z,Z) (Figures 8, 9)with docking characteristics thus: six (6) van der Waal forces, two (2) conventional hydrogen bonds, six (6) alkyl bonds and interacted with these amino acid residues Asn 39, Asp 10, Leu 55, Val 13, Ile 155, Leu 57 and Val 123 at‐5.1 Kcal/mol energy. The hit compound with the lowest binding affinity against TNF‐α, of the three was 9,12,15‐Octadecatrienoic acid (Z,Z,Z) (Figure 9B), which possesses the following binding characteristics: binding energy (−5.0Kcal/mol), five (5) van der Waal forces, one (1) conventional hydrogen bond and seven (7) alkyl bonds. The ligand interacted with the protein via Lys 11, Ile 155, Leu 57, Lys 11, Leu 55, Val 123 and Val 13 amino acid residues.

### 3.4 Absorption distribution metabolism excretion and toxicity (ADMET)/pharmacokinetics predictions analysis of the ligands

Analysis of the hit compounds using Lipinski rule of four is shown in Table 7. In prediction, the potential ADMET profiles of the drug candidates using AdmetSAR server, were determined. The compounds showed different degrees of rule violation, but none violated greater than or equal to (≥) two (2) of the rules and so were considered to possess drug likeness. 9,12,15‐Octadecatrienoic methyl ester (Z,Z,Z) and Hexanedioicacid were found to violate only one Lipinski rule which is that they possess an octanol/water partition coefficient (iLOGP) greater than (>)5 in contrast to the rule which says that it should be less than or equal to (≤) 5, while 6‐Isopropenyl‐4,8a‐dimethyl‐4a,5,67,8,8a‐hexahydro‐1H‐naphthalen‐2‐one and 9,12,15‐Octadecatrienoic acid (Z,Z,Z) violated no rule. The absorption profiles of the hit ligands were also determined and shown in Table 7. The four compounds were all positive for human intestinal and blood brain barrier absorption and negative as P‐glycoprotein substrates. Only 9,12,15‐Octadecatrienoic methyl ester (Z,Z,Z) was positive for P‐glycoprotein inhibition. Moreover, 9,12,15‐Octadecadienoic acid (Z,Z,Z)and Hexanedioic acid were positive for Caco‐2 permeability. The metabolism and toxicity profile of the compounds were also studied and results shown on Table 7. Of the drug candidate compounds 6‐Isopropenyl‐4,8a‐dimethyl‐4a,5,67,8,8a‐hexahydro‐1H‐naphthalen‐2‐one and 9,12,15‐Octadecatrienoic methyl ester (Z,Z,Z) could functionas CYP3A4 substrates;9,12‐Octadecadienoic acid (Z,Z,Z) positive for CYP1A2 inhibition; also, 9,12‐Octadecadienoic acid (Z,Z,Z) and Hexanedioic acid positive as CYP2C9 substrates. Results of toxicity shown on Table 7 indicates that all four (4) compounds were negative for carcinogenicity and hepatotoxicity as well as negative for mutagenicity except 9,12,15‐Octadecatrienoic methyl ester (Z,Z,Z). Moreover, the ligands, excepting 6‐Isopropenyl‐4,8a‐dimethyl‐4a,5,67,8,8a‐hexahydro‐1H‐naphthalen‐2‐one and 9,12,15‐Octadecatrienoic methyl ester (Z,Z,Z) were found to be biodegradable, implying enhance safety profile.

**TABLE 7:** Absorption, Distribution, Metabolism, Excretion and Toxicity (ADMET)/Paharmacokinetic properties of the compounds.

	Orlistat	6‐Isopropenyl‐4,8a‐dimethyl‐4a,5,67,8,8a‐hexahydro‐1H‐naphthalen‐2‐one.	9,12,15‐Octadecatrienoic methyl ester (Z,Z,Z)	9,12,15‐Octadecatrienoic acid (Z,Z,Z)	Hexanedioic acid, bis(2‐ethylhexyl)
Drug likeness					
Mol. Wt	495.73	218.33	392.57	278.43	370.57
LogP	5.46	2.74	5.83	3.36	5.46
HBD	1	0	0	1	0
HBA	5	2	4	2	4
Lipinski violation	1	0	0	1	1
Absorption properties					
HIA	+	+	+	+	+
BBB	+	+	+	+	+
Caco‐2 permeability	−	−	−	+	+
P‐glycoprotein inhibitor	+	−	+	−	−
P‐glycoprotein susbtrate	+	−	−	−	−
Metabolism					
CYP1A2 inhibition	−	−	−	+	−
CYP2C19 inhibition	−	−	−	−	−
CYP2C9 inhibition	−	−	−	−	−
CYP2C9 substate	−	−	−	+	+
CYP2D6 inhibition	−	−	−	−	−
CYP2D6 substrate	−	−	−	−	−
CYP3A4 inhibition	−	−	−	−	−
CYP3A4 substrate	+	+	+	−	−
Toxicity					
AMES toxicity	−	−	+	−	−
Carcinogenicity	−	−	−	−	−
Acute oral toxicity	2.347	2.939	2.639	2.421	2.363
Hepatotoxicity	+	−	−	−	−
Biodegradation	+	−	−	+	+

Abbreviations: BBB, blood brain barrier; HBA, H‐bond acceptors; HBD, H‐bond donors; HIA, human intestinal absorption; Mol. Wt, molecular weight.

## 4 DISCUSSION

The effect of a 6‐week oral administration of *T. conophorum* nut extracts and fractions on the expressions of adipogenesis regulatory target (upstream) gene Peroxisome Proliferator Activated Receptor‐gamma (PPAR‐γ) in the adipose tissue was studied. The data results showed that obesity induction caused a down‐regulation of PPARγ expression in the adipose tissue, but a 6‐week intervention with extract and fractions of African walnut up‐regulated its expression, suggesting a positive modulation of pro‐obesity, pro‐inflammatory and diabetogenic processes/genes, controlled by PPARγ. This observation in the obese subjects agrees with earlier studies where a significant decrease in PPARγ expression was also observed in obese subjects compared to their lean counterparts^22^ In obese condition, adipose tissues undergo dramatic expansion which leads to adipose tissue malfunction. Adipose tissue becomes hypoxic, facilitating chronic inflammatory responses in adipocytes and strongly inhibiting adipogenic differentiation. Such events may lead to epigenetic disturbances such as deregulation of expression of several miRNAs^23^ and DNA methylation which may be involved in deregulating PPARγ expression.^22^ The observed increase in expression levels of the gene following 6 weeks of treatments suggests a protective role against inflammation. This agrees with earlier reports which showed an increase in PPARγ expression and an inhibition of secretion of inflammatory cytokine in patients with chronic obstructive pulmonary disease (COPD) upon treatment with PPARγ ligands such ligands is the n‐3 polyunsaturated fatty acids^24^ present in the African walnut.^11^

The adiponectin expression decreased in obese rats compared to Normal control (NC) and this was similar to earlier reports of adiponectin down‐regulation in obese condition.^25^ Upon treatment with extract and fractions of *T. conophorum* nut for 6 weeks, adiponectin expression was significantly up‐regulated in all treatment groups compared to the obese control. This finding was also in agreement with an earlier report by Qiao, Lee^26^ who showed ‘diets and calorie restriction can dramatically increase adiponectin expression in epididymal fat as well as blood adiponectin levels in both low fat and high fat rodents.’ Consumption of diets rich in fatty acids (omega‐3 fatty acids and eicosapentaenoic acid) as well as carbohydrate diets with low glycemic index and of foods rich in fibre and high fat diet have been shown to increase adiponectin production, and subsequently improved insulin sensitivity, anti atherogenic and anti‐inflammatory effect. Also, consumption of hyperlipidemic diets like cheese, red meat, fried and processed foods rich in saturated fat is also known to reduce the level of adiponectin.^27^ Recently *T. conophorum* nuts were shown to be rich in Omega‐3 fatty acids as well as possess potent anti‐inflammatory properties supporting weight control effect,^11^ therefore, adiponectin gene is another likely molecular target for African walnut in the body weight control mechanism. The effect of the extract and fractions on the expressions of a pro‐inflammatory target genes of the adipose (Leptin and Tumour necrosis factor – alpha, TNF‐α) was evaluated. The result showed that the hitherto up‐regulated TNF‐α expression in the obese rats was deregulated, upon treatment with extract and fractions of *T. conophorum*, particularly the whole ethanol extract treated group. The finding is in line with the research evidence which showed that the adipokine is upregulated in obese conditions. In an earlier study the African walnuts was found to exert anti‐inflammatory properties in MSG‐induced obese subjects.^11^ This ant‐inflammatory action (deregulation of TNF‐alpha gene) may be due to the upregulation of the key genes involved in control of anti‐inflammation processes (like PPARγ and, adiponectin). The PPARγ is known to up‐regulate the adipose‐derived hormone, adiponectin which is famous for its insulin‐sensitizing, anti‐inflammatory and anti‐atherogenic properties.^28^ As observed earlier, increases in PPARγ, was seen to cause corresponding increases in adiponectin expressions for EWE, EAF and RES, respectively. At the same time, PPARγ is also known to downregulate expression of TNF‐alpha^10^; The PPARγ gene is also known to be involved in exerting anti‐diabetic effects by the downregulation of TNF‐alpha, thus inhibiting the induction of progranulin^10^ desensitization of insulin receptors,^29^ while adiponectin enhances insulin sensitivity and increases muscle glucose uptake,^30^ suggesting anti‐diabetic effects of the *T. cononphorum* nut.

Leptin expression was upregulated in obese rats, this was further upregulated upon 6 weeks intervention with ethanol whole extract, ethylacetate fraction and residue of *T.conophorum* nut. This observation is at variance with earlier studies where leptin expression was down‐regulated in diet induced obesity after treatment.^31^ The observed contradiction could derive from the neuro‐degenerative mechanism by which MSG induces obesity as opposed to the simple energy dysregulation mechanism of diet‐induced obesity. Exposure of neonates to MSG severely damages the hypothalamic nuclei which in turn disrupts the hypothalamic signalling cascade of leptin action causing leptin resistance and ultimately leading to overweight/obesity.^32^ This however, should constitute a subject for further research that is, comparing the effect of the African walnut in diet‐induced obese condition, which is more closely related to the human form of obesity with the current report.

Given these impressive molecular data obtained, molecular docking studies were carried out to determine the best in silico interaction of the *T. conophorum* nut derived ligands (active compounds) with the products of the studied genes.

Of the four ligands identified as hit compounds, 6‐Isopropenyl‐4,8a‐dimethyl‐4a,5,67,8,8a‐hexahydro‐1H‐naphthalen‐2‐one is for the first time associated with anti‐inflammatory action *in silico*. These ligands are the likely compounds responsible for the observed molecular effects, hence may be the active agents in African walnut accounting for its anti‐inflammatory action in obesity. Of particular note, is the ligand, 6‐Isopropenyl‐4,8a‐dimethyl‐4a,5,6,7,8,8a‐hexahydro‐1H‐naphthalen‐2‐one, identified as a sesquiterpene lactone,^33^ a derivative of the secondary metabolite *terpenoids* found in plants^34^ which showed high interactions with all the target proteins studied, making it a very promising drug candidate against low‐grade inflammation in obesity.

According to,^35^ the general effect of terpenes is reduction of pro‐inflammatory cytokines such as TNF‐α, IL‐1 and IL‐6. More specifically, sesquiterpene lactones can regulate transcription factors responsible for the release of pro‐inflammatory cytokines (TNF‐α, IL‐1 and IL‐6) and enzymes such as iNOS, COX‐2 etc. and also directly inhibit the release or activity of these mediators.^36^ The detected ligands, 9,12,15‐Octadecatrienoic methyl ester (Z,Z,Z) and 9,12,15‐Octadecatrienoic acid (Z,Z,Z) are derivatives of alpha linolenic acids. Alpha linoleic acid (ALA) is an essential *n*‐3 PUFA with cardio‐protective, anti‐inflammatory and anti‐oxidative effects. Also, ALA or ALA‐enriched diets alter body composition, improve glucose tolerance and attenuate insulin resistance. Taken together, the results suggesting that these ligands may be responsible for the observed molecular actions, hence lead compound for further anti‐obesity research.

No matter how fit a ligand is, with respect to a receptor, it is of no use if it is poorly absorbed, poorly metabolized or poorly excreted from the body.^37^ Hence it was necessary to carry out the ADMET (absorption, distribution, metabolism, excretion and toxicity) evaluation in order to predict the pharmacokinetic (drug‐likeness) profiles of the potential lead compounds. In this study, the ligands did not only have high binding affinities, they also obeyed the Lipinski rule of five, thus, predicting that these compounds will be easily absorbed, as they possessed high permeability profile.

To further buttress this, all hit compounds were found to be positive for human intestinal absorption, denoting that they can be better absorbed from the intestinal tract following oral administration as well as penetrate the blood–brain barrier. None of the ligands are substrates of P‐glycoprotein, unlike the standard drug and so are less likely to be exuded from cells by P‐glycoprotein. The two enzyme isoforms mainly responsible for drug metabolism are P2D6 cytochrome (CYP2D6) and P3A4 cytochrome (CYP3A4).^37^ In this study, all the ligands did not inhibit the CYP2D6 and CYP3A4 enzymes, except the 6‐Isopropenyl‐4,8a‐dimethyl‐4a,5,67,8,8a‐hexahydro‐1H‐naphthalen‐2‐one and 9,12,15‐Octadecatrienoic methyl ester (Z,Z,Z) compounds, which inhibited the CYP3A4 enzyme. It is therefore plausible to predict that the derivatives can be metabolized by the P_450_ enzymes in the body.

Ames toxicity test confirmed that all the test ligands except Hexanedioic acid and 9,12,15‐Octadecatrienoic methyl ester (Z,Z,Z) displayed negative AMES toxicity test similar to the standard drug, while hepatotoxicity test showed that none of the compounds was hepatotoxic unlike the standard drug, suggesting that the ligands are non‐mutagenic and safe for the liver qualifying them for further study as safe anti‐obesity drug candidates.

## 5 CONCLUSION

**FIGURE 10:**
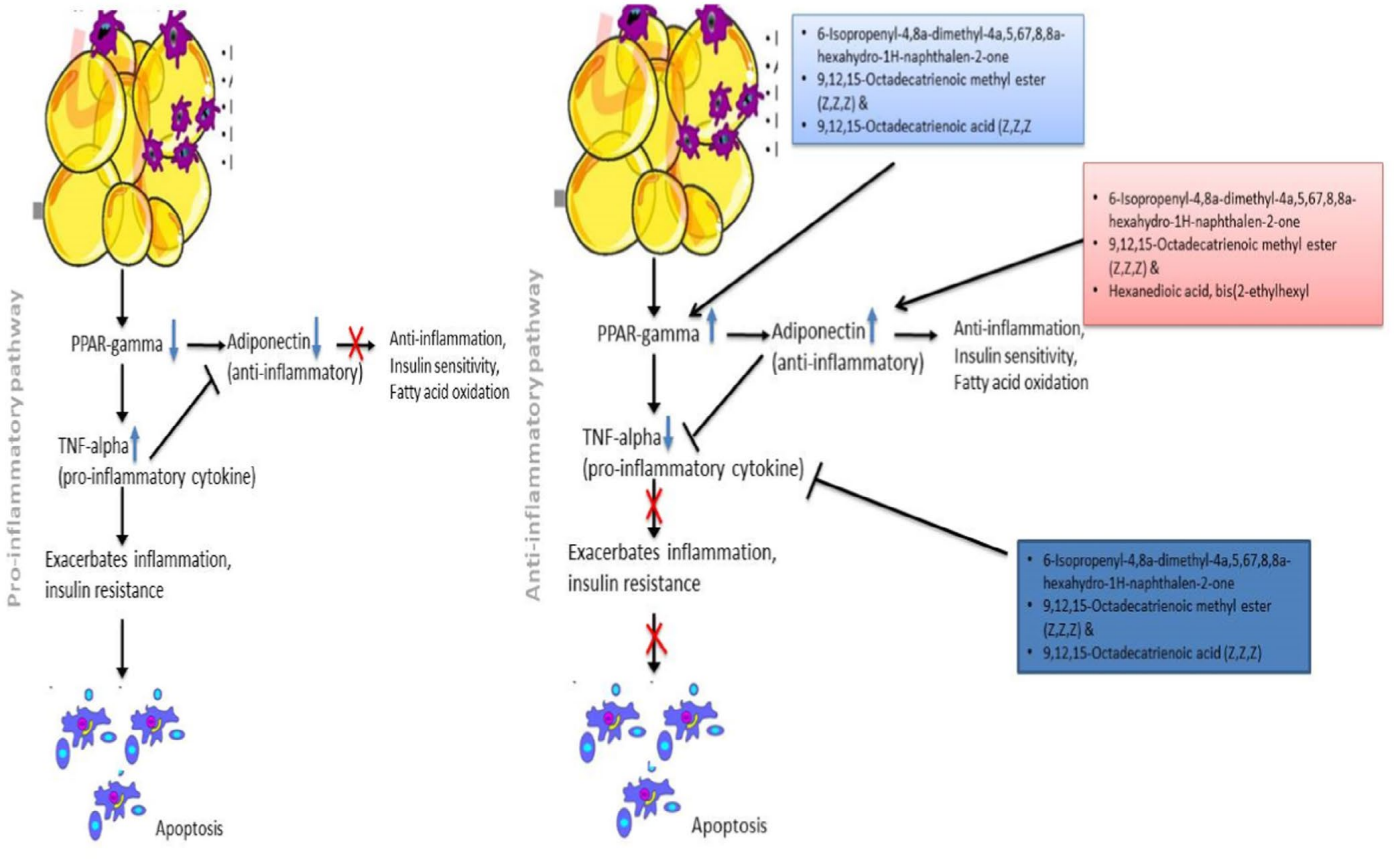
Schematicsummary of the proposed mechanism of anti‐inflammatory action of African walnut.

In total, results from this study point to the ability of the extracts and fractions of *T. conophorum* nuts to protect against inflammation associated with obesity by modulating the activity of upstream/ downstream anti‐inflammatory regulatory genes (PPARG and Adiponectin) and downstream pro‐inflammatory gene (TNF‐alpha) with efficacy in this order: ethanol whole extract > residue > ethyl acetate fraction. And that four active compounds in this nuts namel; 6‐Isopropenyl‐4,8a‐dimethyl‐4a,5,67,8,8a‐hexahydro‐1H‐naphthalen‐2‐one, 9,12,15‐Octadecatrienoic methyl ester (Z,Z,Z), 9,12,15‐Octadecatrienoic acid and Hexanedioic acid, bis(2‐ethylhexyl) are likely responsible for this molecular protection, hence the proposed molecular modulation mechanism on the inflammatory pathway in obese Wistar rats (Figure 10).

African walnuts have been shown to have special bioactive qualities that affect important genes linked to obesity, especially the up‐regulation of PPAR‐γ and adiponectin while down‐regulating TNF‐α, at the molecular level. The therapeutic potential of this alternate regulatory action in managing obesity is noteworthy. The study offers molecular insights into mode African walnuts may affect metabolic pathways, thus: positive attenuation of the obesity‐associated low‐grade inflammation, hence delaying the outset of complication. However, further studies are needed to confirm our findings, particularly, blotting studies with proteins that is, the proteins of the genes studied. The results may pave the way for the discovery of novel therapeutic compounds from African walnuts, in the prevention or management of obesity and its associated metabolic conditions.

## AUTHOR CONTRIBUTIONS

**Grace Ufedo Umoru:** Data curation (equal); investigation (equal); writing – original draft (equal). **Item Justin Atangwho:** Conceptualization (equal); supervision (equal); writing – review and editing (equal). **Esien David‐Oku:** Conceptualization (equal); supervision (equal). **Daniel Ejim Uti:** Data curation (equal); formal analysis (equal); investigation (equal); writing – review and editing (equal). **Eyuwa Ignatius Agwupuye:** Investigation (equal); writing – original draft (equal). **Uket Nta Obeten:** Investigation (equal); validation (equal). **Swastika Maitra:** Formal analysis (equal); validation (equal). **Vetriselvan Subramaniyan:** Formal analysis (equal); validation (equal). **Ling Shing Wong:** Formal analysis (equal); validation (equal). **Nada H. Aljarba:** Formal analysis (equal); validation (equal). **Vinoth Kumarasamy:** Formal analysis (equal); validation (equal); writing – review and editing (equal).

## FUNDING INFORMATION

This work was funded by Princess Nourah bint Abdulrahman University Researchers Supporting Project number (PNURSP2024R62), Princess Nourah bint Abdulrahman University, Riyadh, Saudi Arabia.

## CONFLICT OF INTEREST STATEMENT

The authors declare no conflict of interests.

## Supporting information


Data S1.

## Data Availability

Data is attached to this submission as a supplementary file.
